# Another variant, XBB.1.5: will it be a tsunami for the Americas?

**DOI:** 10.1016/j.lana.2023.100452

**Published:** 2023-02-16

**Authors:** The Lancet Regional Health – Americas

More than 3 years have passed since SARS-CoV-2 first emerged, and although much was done to contain the spread of virus, it was not enough. Applying public health and social measures, developing vaccines, and implementing other measures were crucial. COVID-19 can have major consequences in people's lives by causing debilitating disease, leading to long COVID, affecting mental health, and causing physical sequelae. With more than 6.7 million fatalities over the past 3 years globally, the pandemic cannot be taken lightly. More than 170 thousand people died of COVID-19 globally since January 24, 2023, and the USA is the country with the second highest number of cumulative cases and highest number of deaths in the past week. According to WHO, 323,721 new cases were reported in the previous week. The increase in case numbers is related to the new omicron subvariant, XBB.1.5, that emerged in October, 2022, and spread rapidly, accounting for more than 49.1% of all cases in the USA last week. Most importantly, XBB.1.5 is a cause of concern in that it could partially escape the immune response conferred by the vaccines and previous infections.

Regardless of these concerns, the new subvariant did not spread homogeneously in the USA. It rapidly became the predominant variant in the East coast, but accounts only for 9.5–24.1% of cases in the Midwest and West coast, respectively, depending on the state. According to the WHO report on rapid risk assessment, XBB.1.5 has a moderate risk of antibody escape. Until now, the XBB variants are the most antibody-resistant variants, but there is no known mutation that could influence disease severity. There is the increasing concern that it will soon spread to other countries from the Americas region. The bivalent booster might be a good option to contain the spread of this new variant more efficiently. But it is still not an option for most countries in the region (although in some countries, such as Brazil, it is approved for use, but still not available).

Despite the virus mutation and possibility of evading immune response, vaccination is still the best approach to control the health burden caused by COVID-19. An ecological study using data from a vaccination campaign in Brazil (January, 2021, to January, 2022) has shown that despite the paucity of support by the Government in acquiring vaccines and investing in a proper campaign with support to vaccination, the vaccines averted severe cases and deaths, saving at least 303,129 adults in the country. More patients would have been saved if vaccines were available sooner. In the USA, vaccine availability is not a problem, but coverage is a problem, with only 15.9% vaccinated with the bivalent booster dose. Data shows that there was an increase in hospital admission of children when the omicron variant was most prevalent in the USA, but severe outcomes were fortunately less frequent than with other variants. And although COVID-19 vaccines are approved for children over 6 months old in the USA, this is still not a reality for other countries in the Americas region.

Much needs to be done to prevent disease transmission and excess deaths. According to the Lancet COVID-19 commission, there was a massive global failure with respect to prevention, rationality, transparency, following public health practices, and of operational cooperation and international solidarity. The combination of governmental failure and dissemination of false information was a ticking time bomb that resulted in unnecessary and preventable deaths. Although more than 3 years have passed, governments still need to learn from their mistakes, focus on the prevention of new outbreaks and rebuild public confidence in their measures. Since the beginning of the pandemic, children were not a priority and this needs to change. Countries in the Americas region could start by purchasing and making available bivalent booster vaccines and vaccines for children (aged 6 months to 4 years). Other necessary actions are contingency plans that focus on vaccination campaigns, funding research, acquiring medical supplies, and re-implementing personal protective measures. The XBB.1.5 wave doesn't need to be a tsunami.

